# Supplementary material for: Occurrence of Postoperative Delirium and the Use of Different Assessment Tools
Source: Geriatrics (Basel). 2023 Jan 11;8(1):11. doi: 10.3390/geriatrics8010011 (PMC9844299; doi:10.3390/geriatrics8010011)
Supplement: Supplementary file 1 [file geriatrics-08-00011-s001.zip › geriatrics-2059510-supplementary.pdf]

Table S1. Comparison of visit day and postoperative day for the non-POD and POD group. POD = Postoperative delirium.

**Visit Day 1**				**Visit Day 2**				**Visit Day 3**				**Visit Day 4**				**Visit Day 5**			
**Post-Surgery Day**	**Non-POD**	**POD**	**Sum**	**Post-Surgery Day**	**Non-POD**	**POD**	**Sum**	**Post-Surgery Day**	**Non-POD**	**POD**	**Sum**	**Post-Surgery Day**	**Non-POD**	**POD**	**Sum**	**Post-Surgery Day**	**Non-POD**	**POD**	**Sum**
1	797	78	875	2	780	85	865	3	772	60	832	4	738	51	789	5	697	51	748
2	26	17	43	3	27	15	42	4	34	12	46	5	39	5	44	6	39	3	42
3	8	7	15	4	11	3	14	5	14	3	17	6	13	3	16	7	14	2	16
4	2	3	5	5	2	3	5	6	4	2	6	7	4	1	5	8	3	2	5
5	2	4	6	6	4	2	6	7	1	3	4	8	1	1	2	9	0	1	1
6	1	0	1	7	1	0	1	8	1	0	1	9	2	0	2	10	1	1	2
7	0	1	1	8	0	2	2	9	0	2	2	10	1	2	3	11	1	2	3
9	0	1	1	9	0	1	1	10	0	1	1	13	0	1	1	12	1	0	1
17	0	1	1	10	0	1	1	11	0	0	0	14	0	1	1	13	0	1	1
18	0	1	1	18	0	1	1	13	0	1	1	15	0	1	1	14	1	0	1
Sum	836	113	949	19	0	1	1	14	0	0	0	18	0	0	0	15	0	1	1
				Sum	825	114	939	19	1	0	1	20	1	0	1	16	0	1	1
								22	0	0	0	27	0	0	0	21	1	0	1
								Sum	827	85	911	48	1	0	1	49	1	0	1
												Sum	800	66	866	Sum	759	65	824
